# Year after year: Recurrent *Toxocara vitulorum* infections in American bison (*Bison bison*) calves in a zoo

**DOI:** 10.1016/j.ijppaw.2024.101018

**Published:** 2024-11-08

**Authors:** David Ebmer, Maria Sophia Unterköfler, Zoë Tess Lara Lindhorst, Perrine Keiser, Simone Haderthauer, Stephan Hering-Hagenbeck, Anja Joachim

**Affiliations:** a Vienna Zoo, Maxingstr. 13b, 1130, Vienna, Austria; b Institute of Parasitology, Department of Biological Sciences and Pathobiology, University of Veterinary Medicine Vienna, Veterinaerplatz 1, 1210, Vienna, Austria; c Veterinary Clinic Vienna Zoo, Seckendorff-Gudent-Weg 6, 1130, Vienna, Austria

**Keywords:** Zoological garden, Bovines, Ruminants, Nematodes

## Abstract

*Toxocara vitulorum* (Nematoda: Ascaridida) is a common parasite of cattle and buffaloes in tropical and subtropical regions and the causative agent of toxocarosis in calves. In Europe, sporadic infections have been reported in cattle, but also in bovines held at zoological gardens. Here, we report *T. vitulorum* infections in a herd of American bison (*Bison bison*) kept at the Vienna Zoo, Austria, which occurred in 2023 and 2024. After the first case in a seven-week-old calf in July 2023, another case in a five-week-old calf was diagnosed in May 2024, both of them detected by coproscopy and fecal discharge of adult worms after anthelminthic treatment. The calves originated from two different mothers imported to the zoo in 2014 from the Czech Republic and 2012 from Germany respectively. Both calves showed diarrhea and fecal soiling of the anal region prior to fecal analysis. Two intramuscular administrations of ivermectin (0.2 mg/kg bodyweight, two-week interval) caused the passing of up to 39 cm long gravid female worms, resulted in the cessation of egg shedding and improved fecal consistency. Morphological and molecular identification confirmed infections with *T. vitulorum*. Additionally, another calf, born in May 2024 from the mother of the calf that was *T. vitulorum*-positive in 2023, showed periods of diarrhea. Due to difficulties in taking individual samples, no definitive diagnosis of *T. vitulorum* infection could be made, however, the animal was also treated and clinically improved afterwards. Besides *T. vitulorum*, *Eimeria* spp. were detected in all samples and *Giardia duodenalis* genotype E in two samples in 2024. This case series highlights the possibility of unnoticed parasite introductions into zoological gardens via animals infected with resting parasite stages, and demonstrates the importance of regular individual parasitological analysis in bovine zoo animals during the first weeks after birth.

## Highlights

• American bison calves kept at Vienna Zoo were infected with *Toxocara vitulorum* in 2023 and 2024.
• Two ivermectin treatments (0.2 mg/kg bodyweight) resulted in cessation of egg-shedding and clinical improvement.
• Morphological and molecular identification.

## Graphical abstract

**Figure:**
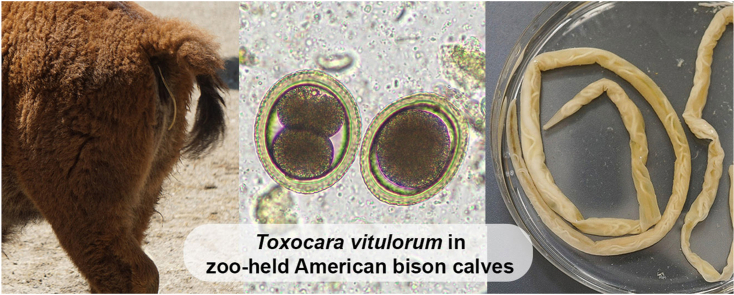

## 1 Introduction

*Toxocara vitulorum* (syn. *Neoascaris vitulorum*) is an ascarid nematode that commonly parasitizes large ruminants (cattle and buffaloes) in tropical and subtropical regions (Rast et al., 2014). Adult nematodes can reach a body length of more than 30 cm and primarily parasitize the small intestine of calves, where they are the causative agents for toxocarosis (Rast et al., 2014), a disease that can take subclinical or clinical courses (Woodbury et al., 2012; Van der Steen et al., 2014; Venjakob et al., 2017) and even result in peracute death (Tscherner, 1991; Schoener et al., 2020). Clinical dimensions depend on the severity of the infection, and especially in endemic areas high morbidity and mortality of calves are reported, additionally associated with economic losses (Rast et al., 2013, 2014).

After ingestion of thick-shelled ascarid eggs containing infective third-stage larvae (L3), larvae hatch and start their somatic (hepato-pulmonary) migration and, finally, enter hypobiotic stages in parenchyma. In gravid female hosts, such larvae become activated shortly before parturition and migrate into the mammary gland (Roberts et al., 1990; Woodbury et al., 2012, 2014). Consequently, *T. vitulorum* is transmitted vertically, via colostrum, from mother cow to calf (Woodbury et al., 2014). The larval burden in the milk is highest in the first days of lactation and strongly decreases after day 9 *post-partum* (Roberts et al., 1990). After ingestion and hepato-pulmonary migration of larvae, pre-adult stages return to the intestines, become mature, mate and the females begin to produce eggs which are shed with the calf's/the host's feces starting two to five weeks after infection (De Souza et al., 2004; Rast et al., 2013).

Oral ingestion of infective eggs by calves is not considered to play an important role in the establishment of patent infections in this age group (Venjakob et al., 2017). Adult bulls are considered as dead-end hosts, since larvae cannot be transmitted to calves (Woodbury et al., 2014). The patent phase is relatively short (usually 5–10 weeks) and calves older than 6 months regularly eliminate *T. vitulorum* adults with their feces (Davila et al., 2010; Woodbury et al., 2014). Patent *Toxocara vitulorum* infections can be diagnosed via coproscopical analysis of egg shedding (Rast et al., 2013; Delling et al., 2020). In addition, molecular and microscopical methods to detect larvae in milk samples are described (Dewair and Bessat, 2020; Urhan et al., 2023).

Hypobiotic stages in infected cows, however, so far evade diagnosis. The infection rates for *T. vitulorum* in buffaloes were 23.0% in a study from Bangladesh (Biswas et al., 2021) and 22.5% in a study from India (Parihar et al., 2022) with the highest rates in the youngest age groups. In addition to reports of this parasite in buffaloes and cattle from tropical and subtropical regions, records were also made in bovines in more temperate regions in Europe, for example cattle in Germany (Venjakob et al., 2017), the Netherlands (Borgsteede et al., 2012), Belgium (Van der Steen et al., 2014) and the UK (Jones et al., 2009). In zoological gardens, *T. vitulorum* has been detected in yak (*Bos mutus grunniensis*) (Fagiolini et al., 2010) and European bison (*Bison bonasus*) (Tscherner, 1991; Delling et al., 2020). In American bison (*Bison bison*), this parasite was detected in farmed animals in Belgium (Goossens et al., 2007) as well as in zoological gardens, e.g. in Berlin, Germany (Tscherner, 1991).

Here, we report *T. vitulorum*-infections in American bison calves in two consecutive years (2023–2024) at Vienna Zoo, Austria, and provide insights into the clinical course, diagnostic methods, and treatment regimes.

## 2 Material and methods

### 2.1 American bison at Vienna Zoo

Since 2015, the American bison herd at Vienna Zoo, Austria, has consisted of a maximum of nine individuals, depending on calving season and animal acquisitions and dispositions. In the first phase of the study, at the end of June 2023, the herd comprised four individuals (one male, two breeding females and one calf, born in April 2023). The first breeding female (internal tag “mother cow no. 1”) was born in April 2010 in Germany and imported to Vienna Zoo in July 2012. The second breeding female (“mother cow no. 2”) was born in June 2013 in the Czech Republic and imported in May 2014. In October 2023, another adult female bison was imported from the Czech Republic. In 2024, two additional female calves were born, one on April 14 to mother cow no. 1, the other one born on May 6 to mother cow no. 2. In the framework of a health monitoring, pooled fecal samples of the herd are regularly (3–4 times per year) analyzed at the parasitology laboratory of the Vienna Zoo. Infections with strongylids, *Trichuris* and *Eimeria*, as well as sporadically occurring infections with *Moniezia* were previously recorded in the herd.

### 2.2 Clinical cases

On June 28, 2023, zoo keepers reported phases of diarrhea and soiled coat in the perianal region of a seven-week-old female calf (originating from mother cow no. 2) and collected an individual fecal sample for further diagnostics. Prior to this episode, clinical signs were not recognized. On May 22, 2024, zoo keepers found diarrheic fecal samples defecated by one or both calves (two females, one born April 14, 2024 to mother cow no. 1, the other one born May 6, 2024 to mother cow no. 2 which already had a positive calf in 2023). Both calves showed fecal soiling in the perianal region and the ventral part of the tail. Despite considerable efforts, however, it was only possible to assign one individual sample to the older calf with certainty. In addition to these samples, individual and pooled samples from the adults in the herd were collected.

### 2.3 Copromicroscopical diagnostics and parasite identification

A combined flotation-sedimentation technique with sugar flotation solution (specific gravity: 1.3) was used for detection of protozoan and metazoan gastrointestinal endoparasites, and stages were graded semiquantitatively as low (+, 1–5 detected eggs/oocysts/cysts per sample), medium (++, 6–10 stages) or high (+++, ≥11 stages). For *Giardia* spp./*Cryptosporidium* spp. detection, coproantigen ELISA-test kits (Megacor, Hörbranz, Austria) were used. Discharged adult parasites were identified according to morphological descriptions (Taira and Fujita, 1991).

### 2.4 Molecular identification

Adult *T. vitulorum* specimens were preserved in ethanol from fecal samples in 2023 and 2024. From a specimen of each year, a 3 mm long piece from the mid-body section was used for DNA extraction. A commercial DNA extraction kit (DNeasy® Blood & Tissue Kit; QIAGEN, Hilden, Germany) was used according to the manufacturer's instructions.

To obtain the barcoding fragment of the mitochondrial cytochrome *c* oxidase subunit I (COI) gene with 654 nucleotide positions, PCR was carried out using primers COI_Nema_fw (5′- GAA AGT TCT AAT CAT AAR GAT ATT GG -3′) and COI_Nema_rv (5′- ACC TCA GGA TGA CCA AAA AAY CAA -3′) (Duscher et al., 2015).

For the detection of *Giardia,* DNA was extracted from the second fecal sample that tested positive in the antigen test in 2024, employing the NucleoSpin® Soil, Mini Kit for DNA (MACHEREY-NAGEL, Düren, Germany) and following the manufacturer's instructions. Three different PCR protocols were carried out by amplifying the glutamate dehydrogenase (*gdh*), triose phosphate isomerase (*tpi*) and β-giardin (*bg*) gene locus (Sulaiman et al., 2003; Cacciò et al., 2008; Lebbad et al., 2010; Tseng et al., 2014; Uiterwijk et al., 2020), each including positive and negative controls.

PCRs were run using GoTaq™ DNA Polymerase (Promega, Madison, WI, USA). The PCR products were subjected to electrophoresis on 2% agarose gels with 4.2 μl Midori Green Advance DNA Stain (NIPPON Genetics, Germany). PCR products of positive samples were sent to Microsynth Austria (Vienna, Austria) for sequencing. The chromatograms were visually inspected and edited using BioEdit (Version 5.0.9) and compared against the NCBI database using BLAST. For the identification of assemblages, sequences were aligned and compared to previously published sequences with known assemblages (Cai et al., 2021; Sprong et al., 2009).

## 3 Results

### 3.1 Copromicroscopy

**Fig. 1:**
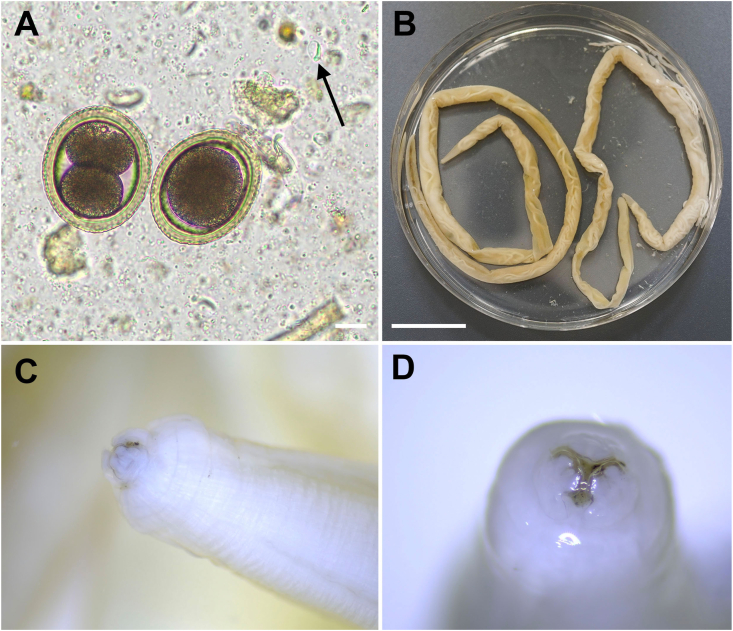
**Morphological identification of *Toxocara vitulorum*.** (A) Two typical thick-shelled *Toxocara* eggs, the left one with the zygote in division to the two-cell stage (Scale bar: 20 μm). A *Giardia* cyst is marked with a black arrow. (B) Two adult gravid females isolated out of the feces (scale bar: 2 cm). (C, D) Anterior parts of a gravid female with the three-lipped mouth parts typical for ascarids.

In June 2023, the individual fecal sample of the seven-week-old calf (originating from mother cow no. 2) revealed high shedding of *Toxocara* eggs (Fig. 1A) and low shedding of *Eimeria* oocysts. Eggs were thick-shelled with a dimpled surface ressembling that of a golfball, and measured 71.8-83.4 × 66.9–76.23 μm.

The day after examination, the calf was treated with ivermectin (Ivomec® 10 mg/ml, Boehringer Ingelheim Animal Health, Ingelheim, Germany) at a dose of 0.2 mg/kg bodyweight intramuscularly (i.m.) via blow-pipe. Calves were not weighed prior to anthelminthic treatment. The weights of the calves were estimated on the basis of records of calves’ weights originating from former breeding and rearing seasons.

A fecal analysis ten days after treatment again revealed positive results for *T. vitulorum* and *Eimeria*. Therefore, another ivermectin treatment was applied, and another fecal control examination ten days after the second treatment returned a negative result for *T. vitulorum*. The fecal consistency clearly improved after the second treatment and no more fecal soiling around the anus was observed after that. Oocyst shedding was still detectable.

**Fig. 2:**
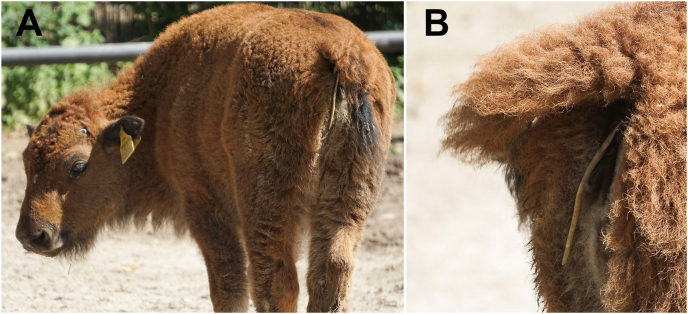
Discharge of gravid adult *T. vitulorum* females after deworming of a bison calf.

In May 2024, a diarrheic fecal sample from one of the calves born in 2024 revealed high-grade *T. vitulorum* egg shedding. Additionally, *Eimeria* oocysts and *Giardia* cysts were detected. The latter was confirmed by a positive fecal antigen ELISA. Both animals were treated with ivermectin as before. The following day, the older calf (originating from mother cow no. 1) shed multiple, large (up to 39 cm long) nematodes which were found hanging from the anus and in the feces (Fig. 2). Fecal samples (not assigned to an individual juvenile) collected five days after treatment were still positive for *T. vitulorum, Giardia* and *Eimeria*; further samples collected seven and ten days after treatment exhibited shedding of *T. vitulorum* eggs and *Eimeria* oocysts but a negative result for *Giardia* (no cysts or antigen detectable). After a second ivermectin treatment, no *T. vitulorum* eggs could be detected in samples taken 10, 15 and 17 days post treatment. Furthermore, no cysts or antigen of *G. duodenalis* s.l. were detected in any of these samples, while *Eimeria* oocysts were still present in all samples. In the following weeks after treatment, clinical signs receded in both calves. In early August 2024, diarrhea was noted in both calves and fecal samples revealed low-grade shedding of *Eimeria* oocysts. Both juveniles were treated orally with diclazuril (1 mg/kg body weight; Dycoxan® 2.5 mg/ml, Chanelle Pharmaceuticals Manufacturing, Loughrea, Ireland). A fecal analysis of adult herd members (pooled and individual samples) revealed negative results for *T. vitulorum* eggs and positive results for *Eimeria* oocysts.

### 3.2 Adult parasite identification

After deworming, multiple fragments of gravid nematodes were detected in fecal samples of the positive calf in 2023. In 2024, a total of five gravid adult females were detected (Fig. 1B–D) in fecal samples and hanging from the infected calf's anus (Fig. 2A and B) after application of ivermectin. Nematodes ranged between 27.2 and 39 cm in length, presented typical three-lipped ascarid mouth-parts (Fig. 1B–D) and could be identified as *T. vitulorum*.

### 3.3 Molecular identification

Both COI sequences were uploaded to BOLD Systems (Process ID: PAVEA257-24, PAVEA258-24) and GenBank (accession numbers: PQ299079, PQ299080). Both sequences had a 100% identity match with each other and with another sequence from *T. vitulorum* found in a yak calf in Austria (GenBank accession number: MK784069). They had 96.8% identity overlap with a *T. vitulorum* sequence from a yak (*Bos mutus*) calf from China (GenBank accession number: NC_070176) and 96.64% with a *T. vitulorum* sequence from a buffalo (*Bubalus arnee*) calf from Sri Lanka (GenBank accession number: FJ664617).

*Giardia* sequences were uploaded to GenBank (accession numbers: *gdh*: PQ310657, *tpi*: PQ310658, *bg*: PQ310659). The sequences obtained at the different genetic loci for *Giardia* all showed a 99.8–100% identity match to the *G. duodenalis* assemblage E found in cattle (*Bos taurus*) and sheep (*Ovis gmelini aries*) in Bangladesh, China, and Greece (GenBank accession numbers: *gdh*: MK982485, *tpi*: MK473860, *bg*: MK862309). All three sequences also aligned best with assemblage E when compared to published sequences.

## 4 Discussion

Here, we report on infections with the large roundworm *T. vitulorum* in zoo-held American bison calves from different mothers in two consecutive years (2023–2024). The introduction of this parasite to the zoo with female bison bred in captivity and transferred as subadult heifers complicates diagnostics - while routine examinations of (sub-)adult herd members remain negative, the parasite can suddenly occur a few weeks after birth following colostral transmission, and patent infections in young sucklers can cause severe disease and even peracute deaths (Tscherner, 1991; Schoener et al., 2020). In this context, we want to raise awareness for this parasite in zoological gardens.

As described previously, in adults, the parasite remains hypobiotic until the onset of lactation. This limits the detection by copromicroscopy to the patent period in young calves. The prepatent period of *T. vitulorum* is reported to be quite variable with two to five weeks of age (Hall, 1999; Rast et al., 2013), while the patent period of around six months is limited to the early suckling period (Davila et al., 2010; Woodbury et al., 2014). In contrast to this, hypobiotic infections in adult animals seem to prevail much longer. The dams of the *T. vitulorum* positive calves were imported to Vienna Zoo more than ten years ago as yearling and two-year old heifer. The cow from the Czech Republic was imported in 2014 and gave birth to a total of seven calves between 2016 and 2024. However, only her offspring of 2023 was detected with a patent *T. vitulorum* infection. For the calf born to this cow in 2024, no definitive evidence for a *T. vitulorum* infection could be established due to difficulties in obtaining individual samples. However, a patent infection is very likely, since the calf also showed symptoms and the mother had already transmitted third-stage larvae via colostrum to her offspring in 2023. Delling et al. (2020) faced a similar problem when they attempted to designate fecal samples to individual European bison calves. The other female with a positive calf was imported in July 2012 from Germany and gave birth to seven calves from 2015 to 2024, but only the last one, born in 2024, was diagnosed with a *T. vitulorum* infection. It is plausible that subclinical cases remained unrecognized for several years in this bison herd. In this context, clinically healthy infected calves could have contributed to a contamination of the enclosure with *T. vitulorum* eggs. Potentially, the environment could have been contaminated with eggs years prior to the entry of both breeding cows. The thick-shelled, environmentally resistant *Toxocara*-eggs can remain infective for several years (Roberts, 1989) and represent a permanent source of (re-)infection for adult, especially female, animals (Venjakob et al., 2017). Analyses of environmental contamination with *Toxocara* eggs were mainly caried out for zoonotic *Toxocara canis and Toxocara cati*, and quantitative determination of environmental egg contamination has been described (Kroten et al., 2016; Tyungu et al., 2020). In the current case, analyses of substrate samples from selected places of the enclosure, e.g. main outdoor enclosure, backstage areas, and stable, could provide information for *T. vitulorum* surveillance in the environment of affected buffaloes in zoos to obtain a better understanding of accumulations of eggs and, consequently, of the level of contamination and infection risk in different areas of the enclosure, to take specific measures for prevention. At Vienna Zoo, the enclosure of the American bison directly neighbors the exhibit of water buffaloes. While transmission between the two species via environmental factors cannot be excluded, no patent infection in buffalo calves have been detected in the zoo up to date. However, monitoring of both species is definitely advisable to avoid a spread of *T. vitulorum* among different bovid species in the zoo and export of parasites with non-patently infected dams to other zoological gardens.

The current cases also demonstrate the importance of individual feces sampling. At Vienna Zoo, no direct contact of bison and zookeepers is possible for safety reasons. Therefore, assigning fecal samples to individuals of the herd is time-consuming and requires intensive animal observations. The example of *T. vitulorum* shows that patent infections can only affect a particular age group of a herd, in this case calves in their first weeks and months after birth (Woodbury et al., 2014). To detect this parasite, examination of pools of several samples taken from the ground are not implicitly representative for the whole herd and present the danger of missing *T. vitulorum* infections in young calves. In the calving season 2022, both mentioned breeding cows gave birth to one calf each, however, no *T. vitulorum* infection could be diagnosed in the herd. It is possible that subclinical infections were overlooked, because fecal samples were sampled during pre-patent periods or did not contain feces of calves of the affected age group.

At the same time, this report exemplifies the importance of regular fecal monitoring in zoological gardens that needs to be adapted to the specific characteristics of different parasitoses. Copromicroscopical analysis of adult animals at the time of their import are not suitable to detect hypobiotic *T. vitulorum* infections that occur in this age class. Thus, *T. vitulorum* can be easily introduced through animal transports and remain unrecognized until its transmission and patent manifestation in suckling calves. At Vienna Zoo, it has to be taken into account that the two dams with positive offspring now represent stable carriers of hypobiotic *T. vitulorum* third-stage larvae that will again be activated at future parturitions. After birth, a close coordination between zookeepers and veterinarians is needed to sample individual feces for detection of infections at an early stage and to ideally treat calves before the onset of clinical signs and shedding of eggs that could serve as a source of infection for adult bison. Interrupting the life cycle of *T. vitulorum* in a zoo appears highly challenging due to the mentioned difficulties in diagnostic evaluations and therefore the parasite must be considered whenever gastrointestinal diseases are observed in animals of the affected age group.

Single intramuscular ivermectin application at the dose recommended for anti-nematode treatment of cattle (0.2 mg/kg bodyweight) was insufficient as it did not terminate egg shedding in either case. However, adult nematodes were already detected in the feces after the first administration, although it was not possible to evaluate the total worm burden, since, especially in the case 2023, only fragments of adult worms were found. After the second application, egg shedding ceased and all control examinations were negative. Subcutaneous application as recommended for cattle was not possible in case of the bison calves due to their shyness and the vigilance of their dams which precluded direct handling; therefore, intramuscular injections via blowpipe were carried out. Similarly, Delling et al. (2020) treated *T. vitulorum* infected European bison calves with doramectin intramuscularly (0.2 mg/kg bodyweight) and reached negative egg shedding periods only after the second treatment period. In naturally infected cattle calves, however, single subcutaneous administrations of ivermectin, doramectin and moxidectin have been reported to be 100% effective after twelve days post treatment and no significant differences between the treatment groups were detected (Avcioglu and Balkaya, 2011). These differences may be due to variations in the pharmacokinetics of macrocyclic lactones in cattle and bison.

With the genetic analysis of the COI barcoding region the morphological determination of *T. vitulorum* was confirmed. The complete identity with a sequence of *T. vitulorum* from Austria (Schoener et al., 2020) and partial identity (<97%) with sequences obtained from *T. vitulorum* from Asia (Wickramasinghe et al., 2009; Xie et al., 2022) may be due to the geographic separation of these cases. The low level of identity indicates a long-term separation of the isolates, but this needs to be confirmed by analyses of worms from different regions of the world.

Besides *T. vitulorum* infections, we detected *Eimeria* infections in the calves in 2023 and 2024. Additionally, *Giardia* cysts and antigens were detected in the first diarrheic sample and right after the first treatment in 2024. *Giardia* was identified as *G. duodenalis* assemblage E (*Giardia bovis*). Previously, genotypes A (*G. duodenalis* sensu stricto, primary host: human) and E (primary host: cattle) were detected in zoo-held bison (Geurden et al., 2009). Surprisingly, only these two samples were positive, and no further *Giardia* detections were made in other fecal samples, neither from juveniles nor from adults. In cattle calves, the pre-patent period of *G. duodenalis* is less than a week, while shedding occurs for at least 60 days (Taminelli et al., 1989). Potentially, an intermittent shedding of cysts or a shortened patent period in American bison might have caused negative results in single samples. Eimeriosis has also been reported as a main causative agent of diarrhea in juvenile American bison (Griffith et al., 2021) and could have contributed to the episodes of diarrhea in the juvenile American bison in this study. Treatment with diclazuril was carried out (at the dose recommended for cattle against *Eimeria bovis* and *E. zuernii*) in August 2024 and fecal consistency improved. Unlike *T. vitulorum*, both *Giardia* and *Eimeria* are transmitted exclusively via the environment, and infection can be detected in the patent phase by fecal examination; however, interruption of the life cycle is difficult due to the low minimal infection doses (Adam, 2001) and the shedding of high numbers of stages by patently infected animals with or without clinical signs (Nydam et al., 2001), so, again, consequent fecal examinations and treatment are necessary for sustainable control.

## 5 Conclusions

We described the sudden occurrence of *T. vitulorum* a few weeks after calving season in a *Bison bison* herd kept at Vienna Zoo. With our findings, we want to sensitize zoo veterinarians for the complex biology of this parasite and call for regular copromicroscopical analyses in large bovines, especially in juvenile animals, to detect these (and other) parasites at an early stage. Retrieving individual fecal samples is time-consuming under the conditions of modern zoo management, but especially important for a parasite whose patent period is focused on a defined host age class. Since zoological gardens constitute ideal institutions to study parasitic diseases in exotic animals, more research on *T. vitulorum* should be conducted to identify the occurrence and prevalence of this nematode. Since infections with hypobiotic larvae cannot be detected due to the lack of egg excretion in these cases, a complete medical history of the group, including potentially diagnosed *T. vitulorum* infections in the past, is essential to assess the infection risk for dams that are transferred between zoos.

## CRediT authorship contribution statement

**David Ebmer:** Writing – review & editing, Writing – original draft, Project administration, Methodology, Investigation, Formal analysis, Conceptualization. **Maria Sophia Unterköfler:** Writing – review & editing, Methodology. **Zoë Tess Lara Lindhorst:** Writing – review & editing, Methodology. **Perrine Keiser:** Writing – review & editing, Methodology. **Simone Haderthauer:** Writing – review & editing, Data curation. **Stephan Hering-Hagenbeck:** Writing – review & editing, Project administration, Funding acquisition. **Anja Joachim:** Writing – review & editing, Writing – original draft, Supervision, Project administration, Conceptualization.

## Ethics approval

All samples analyzed during the study were collected from the environment, and treatments were applied by attending veterinarians as part of the zoo's animal health care program. Vienna Zoo approved all sample-taking procedures, analyses and the conduction of this study.

## Declaration of competing interest

The authors declare that they have no competing interests.
